# The Diagnosis of General Paralysis of the Insane1Read at the 35th annual meeting of the Medical Association of Central New York, held at Syracuse, N. Y., October 21, 1902.

**Published:** 1903-05

**Authors:** William L. Russell

**Affiliations:** Willard, N. Y., First Assistant Physician, Willard State Hospital


### The Diagnosis of General Paralysis of the Insane.[1]

By WILLIAM L. RUSSELL, M. D., Willard, N. Y.,
First Assistant Physician, Willard State Hospital.

AT THE last meeting of the Medical Society of the State of New York, at which no doubt many of you were present, a very able paper on the early diagnosis of paresis was read by Dr. Dercum, of Philadelphia. To some it may seem that a further contribution to this subject, unless to present something entirely new, is at present scarcely called for. I may say, however, that the idea, of presenting this paper was in fact suggested from reading that of Dr. Dercum. His paper discussed only the very earliest stages of the disease, when diagnosis is often extremely difficult. The importance of recognising not only this, but all other forms of mental disease at this period of their development, can hardly be over-estimated, and the writer wishes only to emphasise this importance. By the time cases of general paralysis and in fact, nearly all cases of mental disease are committed to hospitals for the insane, a very long period has elapsed during which an intelligent diagnosis and treatment would have been of inestimable value to the patient, his friends, and to society.

The writer has for several years observed that of the cases of general paralysis admitted to the Willard State Hospital, so far as can be judged from the statements in the medical certificates, and from correspondence with the physicians who signed these, a very few have been diagnosticated before admission. This is not said in a spirit of criticism, but simply as a statement of fact. In most of these cases the disease is well advanced, and the symptoms not at all indefinite. Many of them are recognised as cases of paresis by the nurses who bring them to the hospital. The average practitioner, however, was not, during his student days, trained to make a systematic examination of an insane person, nor to appreciate the diagnostic and prognostic significance of the symptoms presented, and after graduation sufficient opportunities come only to a few. It has seemed to me, therefore, that it might be permissible at this time to supplement Dr. Dercum's paper with one of a more elementary character, in which, while the importance of early diagnosis was emphasised, the salient diagnostic features more or less

---

1. Read at the 35th annual meeting of the Medical Association of Central New York, held at Syracuse, N.Y., October 21, 1902.

common to all stages of the disease might be presented as simply and clearly as possible. Without a knowledge of these, early diagnosis is impossible; with this knowledge one symptom in an obscure case may furnish the required clue.

General paralysis is, unfortunately, at present a hopelessly progressive and incurable disease. If there is ever to be effective treatment, it would seem as though it must be applied at the earliest possible moment, not perhaps months after the patient has first consulted his family physician. Even with the present hopeless outlook, however, and in fact in some instances because of it, there are important reasons why the disease should be recognised by the family physician. The patient needs protection from the effects of his abnormal mental condition. There is sometimes manifested, even in those who in health were temperate and moral, a disposition to excesses, petty crimes, and gross immoralities. Thus, a previously irreproachable character and unblemished reputation, may be irretrievably marred, and the domestic relations of a lifetime broken up, the physical collapse of the patient being at the same time much hastened. The financial interests of the patient, embracing also those of his family and of his business associates, may be jeopardised. He may squander large sums in dissipation, but aside from this, there is an impairment in memory and judgment, which, long before his intimate friends realise the real condition of affairs, may lead to ruinous business mistakes and neglect. Besides, many of the patients whatever their previous disposition, become generous, benevolent, easily imposed upon; and, if in addition to this, as is often the case, delusions of wealth and grandeur be present, even a large fortune may be literally given away. It can be readily understood then, that personal, family, and business interests of the greatest importance may demand that an intelligent diagnosis and the consequent prognosis be made.

In examining a person suspected of mental disease, the same systematic methods should be followed as are customary in making examinations for physical disease. Hereditary tendencies should be inquired into. The main facts concerning the life and conduct of the patient from childhood up should be ascertained as far as possible. The history of the present illness from its earliest inception up to the time of the examination should be obtained, and the salient features put together and scrutinised as a whole. A minute physical examination should always be made, if the condition of the patient permits. In the course of such an investigation as this, the possibilities, and then the probabilities as to nature of the disease may be very early suggested. The examiner must then follow the various clues and discover where they lead. In weighing the probabilities as they may relate to general paralysis, there are certain etiological factors, which the examiner will do well to keep in mind.

1. General paralysis is a disease of modern civilisation. It is practically unknown among the uncivilised, and scarcely can be said to exist in the more remote rural districts of civilised countries. It is met with more frequently as the vicinity of towns and cities is approached, and reaches its maximum prevalence in the larger centers. The disease affects all classes of society. It is most frequently met with in persons of considerable activity, physical, mental, or both, especially those who work hard and at the same time because, of dissipation or for other reasons, do not take sufficient rest to recuperate their exhausted nervous system.

2. Absence of hereditary tendency to mental and nervous diseases, is more frequently observed in cases of general paralysis than in other forms of mental disease.

3. General paralysis is at least four times as frequent in the male sex as in the female.

4. The large majority of the cases occur between the ages of 35 and 45, the average age being about 38. The disease, however, may appear at any age. There have been a few instances in children, the victims of hereditary syphilis, and it is not infrequently met with in persons over 50, and even over 60.

5. The relation of alcohol to general paralysis is still unsettled. It is safe to say, however, that it is more frequently found in those given to dissipation of all kinds. It is occasionally seen apparently as a result of lead poisoning.

6. That syphilis is one of the most potent factors in the causation of general paralysis, modern authorities are agreed. Krafft-Ebing goes so far as to refer to the disease as the product of civilisation and syphilisation, and there is a strong inclination on the part of many to look upon syphilis as the essential causative agent. A history of syphilitic infection in the individual would therefore be suggestive. The difficulty of obtaining accurate information on this subject is, however, well known. It is possible for a person to be ignorant of his infection. There is also nearly always an inclination to concealment. In relation to general paralysis, not only acquired but hereditary syphilis must be considered, and the difficulties are further increased by the fact that the statements of the sufferer from mental disease are often entirely unreliable; and as there may have been no active signs of syphilis for the preceding ten or twenty years, the friends may be unable to give any information whatever, or may be led to make an unwarranted denial.

Given, therefore, a male patient of the proper age with a progressive mental disorder however mild, accompanied by general nervous and muscular disturbances however vague, whose history indicates previous good health and activity with special freedom from such neuroses as neurasthenia, hysteria, and the like, with an inclination toward a strenuous or fast life in business or pleasure, who has inherited no special tendency toward nervous or mental disease, and who has several years previously been infected with syphilis, or whose habits and mode of life afford ground for suspicion of infection, and the presumptive evidence is strongly in favor of general paralysis. The conditions are, however, rarely so nearly typical. The patient may be a woman. In such cases she usually belongs to the uneducated classes. She is almost invariably married or else a prostitute. Otherwise, the points to be considered are the same as in the case of a male. A hereditary tendency to neuroses or insanity may be discovered. This is by no means uncommon and, in fact, some observers claim to have found it in the majority of their cases. The patient may be older or younger than the ages at which the disease prevails most. No suggestion nor suspicion of syphilitic infection may be obtainable. And yet, upon examination, it may still be found that there are reasons for suspecting general paralysis. In such cases, the weight to be attached to the information obtained will depend upon the history of the present illness, and the results of the personal examination of the patient.

The early history of general paralysis has been well described in Dr. Dercum's paper, as are also the differential points by which the disease may be distinguished from neurasthenia, the condition for which it is most likely to be at first mistaken. As explained by Dr. Dercum, the distinction can in most cases be made by bearing in mind the clear and well-defined symptomatology of chronic fatigue—neurasthenia—on the one hand, and the special symptoms of the mental weakness and degeneration of paresis on the other. It sometimes happens that the history gathered by the examiner may be alone sufficient to establish the diagnosis, the personal examination simply confirming what was practically already a foregone conclusion. Often, however, enough only is obtained to suggest the possibility of the disease. The details must then be gathered by investigation and pieced together. In this process the point to be especially kept in mind is that the characteristics of the clinical picture of general paralysis, is the *concomitant* appearance of mental and physical symptoms. On the mental side there is progressive dementia, to which is generally superadded insanity of the maniacal, melancholic, or confusional types; on the physical side there is paresis and incoördination of certain parts of the motor mechanism, with partial degeneration of the osseous, cartilaginous, and muscular tissues. (MacPherson.)

The history presented usually is one of gradual physical and mental deterioration. Sometimes, however, the first notice of the disease has been an epileptiform convulsion. Such an occurrence in a person presenting, in any degree, favorable etiological conditions should arouse the gravest suspicion. Or the occasion for seeking medical aid may be an acute maniacal outbreak. In such cases the most careful examination may be insufficient to reveal the real nature of the disease. Even at the hospital they are sometimes at first classified as acute mania, and a favorable prognosis given. A fuller history of the beginning of the illness, such as can more frequently be obtained by the family physician, would probably prevent this error. When insanity of a melancholic or confusional type is present, mistakes are less likely to be made, clearer evidence being usually obtainable on personal examination of the patient. The history obtained by the examiner is, however, usually somewhat indefinite. The patient has probably been noticed to be generally failing in health. He has had digestive disturbances, headache, neuralgia, vague muscular pains, lassitude and disinclination to exertion. His movements are noticed to be more uncertain. If a mechanic, he has lost his dexterity in the use of tools, he cuts himself, he botches his work; if a business or professional man he cannot write so well, he fumbles his knife and fork, he loses his skill at mechanical games. Mentally, there may be a history of change in disposition, habits, and character, with depression of spirits and hypochondriacal notions, or there may be elation and a sense of well-being with expansive ideas. Whatever the history may be, the examiner, his suspicions once aroused, should seek by means of questioning and by personal examination to elicit the essential physical and mental features of the disease. These are, on the physical side:

1. Progressive incoördination and paresis of all or of portions of the general muscular system. At first, these may be scarcely noticeable. Or, they may appear only when the patient attempts to make finely coördinated movements. Or, they may render the figure slouchy and the gait shuffling and uncertain. Eventually they render the patient bedfast and helpless. A striking feature is relaxation of the muscles of expression, rendering the face smooth, mask-like, expressionless.

2. Tremor.—This is often noticeable very early. It affects principally the lips, tongue and muscles of expression, but eventually is noticeable throughout the voluntary muscular system. The patient should be asked to write and execute other movements requiring steadiness and certainty of movement.

3. Embarrassment of speech is the most prominent physical symptom.—It should always be looked for and when found should be given great importance. To be thoroughly appreciated and readily recognised it must be heard. The first sign of speech disturbance is hesitancy in attempting a more than ordinarily difficult word. The patient pauses, makes a bad attempt at pronunciation, followed possibly by a successful repetition. The friends of the patient may say that he lisps or stumbles in his speech. The examiner may notice the difficulty in pronouncing certain letters, or a tendency to slowness, and the careful, stacatto pronunciation of each syllable. Little may be observable in ordinary conversation, and it is customary to ask the patient to pronounce certain test words. Among these are "communicability," "truly rural," "artillery," "peach tree creek," "Methodist-Episcopal."

4. Pupillary Anomalies.—These are among the most common and significant symptoms of the disease, though they vary in different cases. Inequality of the pupils is most frequently seen, the more dilated pupil being the affected one. The pupils may be markedly contracted and irresponsive to light or accommodation, or they may react to accommodation and not to light. Irregular contour of one or both pupils is not infrequently met with. In the later stages of the disease there is a tendency to dilatation of the pupils.

5. Disturbances of the Reflexes.—The knee-jerk is usually exaggerated, but may be normal. The superficial reflexes are, as a rule, lowered or diminished.

6. Sensory Disorders.—There is usually progressive impairment of cutaneous sensibilities, with a loss of the feeling of pain, and of the thermal sense. Analgesia of the ulnar nerve is considered by some a diagnostic symptom of general paralysis. Tests should be made especially on the chest in front, the lower limb, the upper limb, the face, and hands.

7. Trophic changes.—There is a marked interference with the nutrition of the body. At first there is a tendency to loss of flesh, and the patient looks haggard and fatigued, though later as the dementia deepens he may become quite corpulent. The skin loses its normal texture and color, the nails become rough, fissured, and the hair dry, brittle, and stubborn. Later in the disease more profound trophic changes appear.

8. Epileptiform and Apoplectiform Seizures.—These may occur quite early in the course of the disease, and as indicated may be of great diagnostic importance. They may, on the other hand, be absent until quite late. They are sometimes followed by paralyses which are often transient.

The signs of locomotor ataxia are frequently met with in cases of general paralysis, the two diseases being sometimes associated. In fact, the pathologic process is the same in both instances, the symptoms depending upon the location of the changes in the nervous system.

There is but one invariable mental symptom in the clinical history of general paralysis,—mental weakness. The history related to the examiner may indicate depression of spirits and painful thoughts or, as more frequently happens, mild elation and expansive ideas. There may be positive maniacal excitement with a tendency to destructiveness and violence, or pronounced melancholia with suicidal inclinations. There may be the most extravagant delusions of wealth and grandeur, or of danger of bodily injury or disease. None of these is distinctive, not even delusions of grandeur, which are by many, not practically familiar with the disease, considered essential to the diagnosis. The gay, confident manner and boastful extravagant language of a certain common type of paretic, with the characteristic countenance, and embarrassment of speech, present, indeed, a striking picture which once seen and appreciated can ever after be recognised at a glance. All these, however, may be lacking, especially in the earlier stages of the disease. The key to diagnosis is the discovery of definite mental weakness in connection with the physical signs. The loss of the sense of propriety and of other finer feelings, of will-power, and of self-control, with consequent predominance of the animal propensities; the failure in business judgment and foresight; the foolish, extravagant, childish conduct; and the lapses of memory which have been noticed by friends and associates are evidences of this weakness.

When the history is indefinite the examiner may himself be able to elicit the required evidence. The conduct and conversation of the patient, while in his presence, may themselves show his poor emotional control, and his incapacity for sustained attention and thought. A few specific questions in regard to remote and recent occurrences with which he ought to be familiar may disclose decided failure in memory, and abnormal indifference and inattention. The simpler these questions, and the more definite the answers required, the better. The hour, day of the week and month, the month and year, the date of birth and marriage, the names of prominent persons and of friends, routes of travel from place to place, the market prices and important newspaper items, are examples of suitable subjects. Simple problems in mental arithmetic may be given to test the patient's computing power, which is almost invariably impaired even quite early in the course of disease. The education, occupation, and station in life of the patient will, of course, have to be considered in estimating the value of the supposed evidences of mental weakness.

Permit me in closing to emphasise the following points: (1) the necessity and importance of recognition, by the family physician, of general paralysis and other forms of mental disease in their early stages; (2) the relation of modern civilisation to the prevalence of general paralysis; (3) the relation of syphilis as a causative factor; (4) that the characteristic of general paralysis is the concomitant appearance of physical and mental symptoms; (5) that the physical signs are definite, characteristic, and, as a rule, easily discoverable; (6) that the essential feature of the mental condition is weakness.

WILLARD, N. Y.

---

THE severe pain of gout has been promptly relieved by the application of lint saturated with alcohol and covered with oiled silk.—*Canada Lancet.*

